# The Uses of Sanatoria for the Treatment of Tuberculosis

**Published:** 1901-03

**Authors:** C. Braine-Hartnell

**Affiliations:** Resident Physician to the Cotswold Sanatorium.


### THE USES OF SANATORIA FOR THE TREATMENT OF TUBERCULOSIS.

BY

C. Braine-Hartnell, M.R.C.S., L.R.C.P.,

*Resident Physician to the Cotswold Sanatorium.*

---

A remarkable wave of feeling in favour of open-air treatment has passed through the country during the last few years, uprooting prejudice, and setting aside old-timed ideas. This feeling was undoubtedly generated by spirited articles in *The Practitioner*, nurtured in the arms of other medical and lay papers, and brought to maturity by the formation of various sanatoria for well-to-do patients throughout the land. That the idea was not the exaggerated dream of an enthusiast is shown by the fact that it is not ephemeral in character, but is standing adverse criticism, and that which is greater than all criticism—the test of time.

Ten years ago sanatorium treatment was practically not mentioned in our medical schools, whilst to-day it is a common topic of conversation. Three years back, with one or two exceptions, there were no sanatoria in the British Isles, and yet to-day there are over thirty in full working order. We would ask why there should be this sudden up-springing of sanatoria unless this treatment were a success? Those who deny the good results obtained in sanatoria speak in ignorance, because they do not know. We do not claim that sanatorium treatment is a panacea for every tubercular ailment, and that every case, however far advanced, will be benefited by such treatment. Far from it. We know only too well our limitations; but this we do claim, that it gives far better results than any other method of treatment that we know as yet. Let it once be allowed that pulmonary tuberculosis is curable, and that under ordinary circumstances and without undue care, then is it too much to assert that arrest and obsolescence of the disease can and do take place in sanatoria, where patients are under constant supervision and where everything is made subservient to the all important idea of getting well.

Hippocrates held the view that, if a "consumptive" patient be treated from the beginning, he will get well. He advised exercise and rest, the two important points in the treatment of to-day. Pliny, Celsus, Galen, and others, also looked upon tuberculosis as curable. In fact nothing is more interesting than to read the history of tuberculosis, and to see how firmly this belief was held in bygone ages. Carswell wrote in 1838: "Pathological anatomy has never given more decisive proof of the curability of a disease than it has given for pulmonary consumption." This point cannot be disputed.

Healed tubercular lesions have been found in varying proportions in persons dying from some other disease.

| | | | | |
|---|---|---|---|---|
| Ormerod | found arrest in | 4 | per cent. | of cases. |
| Kingston Fowler | ,, | 9 | ,, | ,, |
| Sidney Martin | ,, | 9 | ,, | ,, |
| Heitler (Vienna) | ,, | 4 | ,, | ,, |
| Fürbringer (Berlin) | ,, | 10 | ,, | ,, |
| Osler | ,, | 7 | ,, | ,, |
| Coats | ,, | 4 | ,, | ,, |

That great master of clinical medicine, Charcot, wrote that phthisis is susceptible of being cured completely and definitely, even at the period of cavitation.

Practically every country in Europe boasts of sanatoria. In Germany they have existed for over forty years; there is one in connection with most large towns, and insurance companies are even building sanatoria in which their own policy-holders can be treated when the disease has been detected. America has also many excellent sanatoria. In fact in the British Isles we are far behind almost every other country, and we are still asking one another whether the treatment can possibly be carried out in this country with any success, and whether the craze will last.

Let us say at once that we believe that the treatment can for the majority of people be quite well carried out in England, Scotland, or Ireland, and with excellent results. We do not say that climate has no effect on the disease, but that climate has very much less effect than most people think, and given a good soil and good protection from winds, we do not care much for climate; in fact we think that an Englishmen can be cured in his own country, and that he should be if he intends to live in it afterwards. We believe most strongly that cases that recover in Switzerland, Canada, The Cape, or Australia, can recover equally well in England. Cases that react to the treatment in one country can react to it in another under suitable circumstances. We put the matter strongly, because patients have done well, and will do well, in British sanatoria under strict supervision, and we think that the time has now come to erect in this country sanatoria for the poorer classes. It is not a leap into the unknown, it is simply following where others lead. The open-air treatment is no fad, it is recognised as *the* treatment of the day.

The questions may arise—What is the use of Sanatoria? and, Cannot patients be treated at home? We would answer the second question first, and that very decidedly in the negative. It would be difficult to treat well-to-do patients in country houses, but it would be absolutely impossible to treat the poor in their cottages in large towns. This is only in accordance with common sense. The treatment consists in: 1. Constant supervision. 2. Fresh air. 3. Rest. 4. Exercise. 5. Food (in abundance). Food is no good without fresh air; exercise is dangerous without medical supervision. It is the combination of all these points that leads to success; one merges imperceptibly into the other; take one away and the whole treatment is spoilt. It is obvious that the treatment can only be thoroughly carried out in a sanatorium; to attempt to carry it out at home without being educated in a sanatorium is to bring discredit on a good system. Secondly, to deal with the uses of a sanatorium. The greatest use, in our opinion, is, that it is a school for discipline and education. For discipline, because patients have to keep regular hours, and to fall in with the rules of an institution; and as the majority of patients are young, this discipline is doubly urgent. For education, because they have to learn: 1. That the disease is infectious, that they must not spit here, there, and everywhere, but in the proper pocket-flasks, and that sputum must not be swallowed; and thus dissemination of disease is prevented and auto-infection reduced to a minimum. 2. That all winds must be avoided, and consequently that walks must be chosen according to the wind. 3. That no exercise must be taken while the temperature is raised. 4. That all violent exercise must be given up, such as dumb-bells and rowing. 5. That there must be rest before meals. 6. That the whole twenty-four hours must be spent in the open air. The educational side is most important, as few patients can afford to stay in a sanatorium until they are well, nor indeed is this necessary. Educate patients to an open-air life and then they can carry it out in the country, but do not try to carry out treatment in a country house, until your patients have been educated in a sanatorium. Three months in a sanatorium will send patients home with air-hunger; they will be unable to live without open windows; they will have no fear of night air, and a rainy day will not keep them indoors; they will no longer be a source of infection to their friends, and they will know how to carry on treatment.

How long is it necessary to live in a sanatorium? The longer the better. Do not send advanced cases and expect, as some people do, to find them well in three months; it is expecting what is impossible. *Nobody is cured in three months.* The disease may be arrested in a few cases, but not cured. A cure is a case that can stand the test of time. If a patient has no physical signs, no temperature, no cough or expectoration, and if he keeps up this condition for two or three years, then and only then can he be called cured. Anything less than this may be a complete arrest, but it is not a cure. It is a mistake to expect miracles to be wrought in a sanatorium. There may be, and are, excellent recoveries, but these are all the result of careful supervision spread over many months. Advanced cases require from eighteen months to two years for recovery. If all patients were told this, there would be fewer disappointments because they were not cured at the end of twelve weeks. In the face of this it seems to us imperative that only incipient cases should be sent to sanatoria for the poorer classes; to send advanced cases is to court failure. It may seem hard, but we are sure it is wise: the old saying, that a prognosis depends to a great extent upon a man's means, still holds good. The man who can live in a sanatorium for twelve months, and then spend the rest of his life, or at any rate two or three years, as a country gentleman, must necessarily stand a better chance of getting well than a poor man who, after three to four months' treatment, has to go back to an ill-ventilated, badly-drained, sunless cottage in the centre of some big town.

The results of sanatorium treatment are very gratifying. Within a very short time weight is put on—often three to five pounds per week for the first few weeks, the temperature falls, the night sweats are lost, the appetite improves, the complexion becomes healthy, the cough and expectoration are lessened. An increase in weight by itself is worthless and misleading; it is to be regarded with caution unless accompanied by some other good sign, such as improved complexion (which is very important) or a falling temperature. Here again we would lay great stress upon the importance of taking a *rectal* temperature. It is one of the secrets of success, as in many cases both the oral and axillary temperatures are misleading, scarcely ever rising above normal, whilst the rectal record shows at once the slightest activity.

The question is often asked—Will any treatment such as a serum or an antitoxin in time to come supersede sanatorium treatment? We believe not, as we consider that, for any treatment to be successful, the general health must be good, and this is best achieved in a sanatorium. Any antitoxin treatment will simply be carried out in conjunction with sanatorium treatment, and even then will be a work of time.

We repeat that the open-air treatment in a sanatorium is the best treatment yet known, and it gives such good results that it is our duty as a philanthropic nation to put this treatment within the reach of our poorer brethren, by building sanatoria into which they can be admitted free of charge.

